# Systematic review on the use of foot orthosis in symptomatic pes planus

**DOI:** 10.1186/1757-1146-5-S1-P2

**Published:** 2012-04-10

**Authors:** Helen A Banwell, Shylie Mackintosh, Dominic Thewlis

**Affiliations:** 1 School of Health Sciences, University of South Australia, Adelaide, South Australia, 5001, Australia

## Background

Physiologic (flexible) pes planus (flatfoot) is a descriptive term for feet that have a visually lowered medial longitudinal arch often in association with rearfoot eversion [1]. Reported to affect approximately 15% of the adult population [2] physiologic pes planus can be categorised as either symptomatic (painful, non-functional) or non-symptomatic (non-painful, functional) with the literature purporting that flexible non-symptomatic pes planus is a predominantly benign condition with no justification for intervention [3]. When pes planus is symptomatic however, functional foot orthoses are often prescribed and are the most commonly quoted intervention within the literature[4]. Despite some controversy for their use in pes planus, functional foot orthoses remain the cornerstone of podiatric management of this common disorder. The aim of this review is to evaluate the evidence for the use of foot orthoses in adults with symptomatic pes planus.

## Materials and methods

A systematic review of randomised, quasi-randomised or controlled clinical trials comparing rigid or semi-rigid functional foot orthoses with: no orthoses or any other approach to managing symptomatic flexible pes planus in the adult. Relevant data bases were searched from inception to October 2011 with studies meeting the predetermined inclusion criteria retrieved and screened by two independent reviewers. No restrictions were placed on type of foot orthoses or alternative interventions. Included studies were independently assessed by two reviewers with risk of bias determined by the Cochrane criteria. Where feasible, meta-analysis will be undertaken.

## Results

A narrative summary will be presented of the results with outcome measures to include: pain, gait changes (efficiency, temporal or spatial changes), dynamic foot function or static foot posture changes.

## Conclusion

A determination of the current level/s of evidence for the use of foot orthoses in the adult with symptomatic pes planus.
